# Mycetoma Herbal Treatment: The Mycetoma Research Centre, Sudan Experience

**DOI:** 10.1371/journal.pntd.0002400

**Published:** 2013-08-22

**Authors:** Eshraga A. Ezaldeen, Ahmed Hassan Fahal, Anjom Osman

**Affiliations:** Mycetoma Research Centre, University of Khartoum, Khartoum, Sudan; University of Tennessee, United States of America

## Abstract

It is still challenging and difficult to treat patients with eumycetoma; the current treatment has many side effects and has proven to be expensive and characterized by high recurrence rate, hence the poor patients' treatment compliance. Most of the patients are of low socio-economic status, have many financial constraints and hence, many of them rely on alternative and herbal medicine for the treatment of their disease. With this background, the current study was conducted to determine the prevalence of herbal medicine usage among patients with eumycetoma. This cross-sectional, observational, questionnaire-based study was conducted at the Mycetoma Research Center, University of Khartoum, Khartoum, Sudan. A convenience cohort of 311 patients with confirmed eumycetoma was invited to participate in the study after informed consent. The study showed that 42.4% of the study population used herbal medicine for the treatment of eumycetoma at some stage of their illness. The commonly used herbs were *Moringa oleifera*, *Acacia nilotica*, *Citrullus colocynthis* and *Cuminum cyminum*. Most of the patients claimed no benefits from the herbal treatment. Ninety one patients (29.3%) had encountered complications with herbal treatment. The high prevalence of herbal treatment encountered in the study can be explained by the patients' dissatisfaction with the current medical therapeutic modalities. To reduce the high prevalence of herbal medicine usage, governmental control and health policies are mandatory; likewise, native healers need to be educated in that. *Moringa oleifera* was the commonly used herb in this study and many reports claimed medicinal properties of this tree; hence, further in-depth studies to determine the active ingredients in the different parts of the tree and its effect are required.

## Author Summary

This work was conducted to determine the use of herbal treatment among a group of eumycetoma patients. As mycetoma is a good example of a badly neglected tropical disease across the world, many mycetoma patients tend to present late with advanced infection, one of the important reasons for which is the use of herbal medicine for quite a long time; hence, this study was conducted to determine that. The study showed that a good number of the patients used it, many of them not aware of the type of herbs used. This treatment was associated with many complications. Raising the patients' and community's awareness on the negative impacts of this treatment is important to decrease its use and complications and to encourage patients to present early for medical treatment.

## Introduction

Eumycetoma is a common medical and health problem in the tropical and subtropical subcontinent [1]. It is a chronic specific granulomatous subcutaneous disfiguring and disabling inflammatory disease. *Madurella mycetomatis* is the main causative fungal agent responsible for eumycetoma in various parts of world [2], [3]. The triad of a painless subcutaneous mass, sinuses formation and purulent or sero-purulent discharge that contains grains is pathognomonic of mycetoma [4], [5]. The disease usually spread to involve the skin and the deep structures, resulting in destruction, deformity and loss of function, and occasionally it can be fatal [6]. Mycetoma has many serious health and socio-economic impacts on patients, families, communities and health authorities [1], [2].

The current standard treatment of eumycetoma is a combination of antifungal drugs and surgical excisions [7]. The antifungal in use presently are ketoconazole or itraconazole, they proved to have many adverse reactions, expensive and associated by high recurrence rate [8], [9]. The surgical excision ranges from wide local excision, repeated debridement and amputations [9]. In general, treatment of eumycetoma is associated with many side effects, high recurrence and follow up dropout up rates, disfigurement and disabilities [10].

The WHO has recently defined traditional medicine and herbal medicine as the therapeutic practices that have been in existence, often for hundreds of years, before the development and spread of modern medicine and are still in use today [11]. Herbal treatment primarily uses medicinal plant preparations for therapy. In Sudan, herbal treatment for various medical problems is a common practice (Unpublished data). That can be explained by the massive lack of health education, poor medical and health facilities in remote areas, patients' poor socio-economic status as well the availability of many traditional healers in the local communities. In mycetoma, the patients are generally dissatisfied with the available treatment in addition to the other mentioned facts.

*Moringa oleifera* which is a tropical tree, with various medicinal properties, recently gained popularity as a herb for the treatment of many medical conditions in the Sudan [12].

Many herbs are in use for mycetoma and they vary from one community to the other. To determine the prevalence of herbal treatment among patients seen at the MRC, this cross- sectional study was set out and conducted.

## Materials and Methods

This observational, cross-sectional questionnaire based study was conducted at the Mycetoma Research Centre, Khartoum Sudan, in the period November 2012 to February 2013. A convenience cohort of 311 patients with confirmed mycetoma was invited to participate in the study.

The sample size was calculated from the total population of 6200 patients registered with the MRC, and the confidence level of 97% and the limit of precision of 6% were used. As no similar study was reported previously on the prevalence of mycetoma herbal treatment, 50% herbal treatment prevalence was used.

A structured questionnaire was used; it included the patients' demographic characteristics, information on the used herbal treatment and patients' knowledge on mycetoma.

The collected data was managed by SPSS computer programme (SPSS-20). Descriptive analysis and cross tabulation were done. Chi-square tests were used to test association. Level of significance was set as *p* value<0.05.

### Ethics Statement

Written informed consents were obtained from all the study participants and ethical clearance was obtained from the Ethical Clearance Committee of Soba University Hospital.

## Results

The study included 311 patients with confirmed eumycetoma, 241 (77.5%) were males and 70 (22.5%) were females. Their ages ranged between18 and 72 years (mean 32±5 years); 244 (78.5%) were in the age group 18–37 years, 56(18%) were in the age group 38–57 years and 11(3.5%) in the age group 58–77 years.

In this study, 132 patients (42.4%) had history of herbal treatment at some stage of their illness while 179 patients (57.6%) had no history of such treatment. The demographic characteristics of the two groups are shown in Table 1.

**Table 1:** The demographic characteristics of the study population.

Characteristic	Herbal Patients	Non-Herbal Patients
No.	132	179
Gender		
Male	97	144
Female	35	35
Mean Age	32 years	31 years (p = 0.6)
Mean Disease Duration	91 Months	77 Months (p = 0.037)
Occupation:		
Students	27	34
Farmers	24	27
Workers	21	41
Other	60	77
Education Level		
No formal education	19	34
Primary school	42	54
Secondary school	54	57
University	17	34 (p = 0.3)

The patients used different herbs types; either as a single herb or as a combination of different types. The commonly used herbs were *Moringa oleifera* (18.9%), *Acacia nilotica* (9.8%), *Cuminum cyminum* (5.3%) *Citrullus colocynthis* (4.5%). (Table 2). Twenty three patients (17.4%) used mixture of herbs; 18 patients (13.6%) used *Moringa oleifera* combined with other herbs and five patients (3.8%) used Cuminum cyminum combined with other herbs. The prevalence of using *Moringa oleifera* in this study was 18.9%. (Table 2) in this study, sixty six patients (50%) didn't recognize the used herb type or its ingredients, and 18 patients (13.6%) used wide range of different other herbs. (Table 2)

**Table 2:** The prevalence of different herbs used among the study population.

The Herb Medicine	Patients No.	%
***Moringa oleifera***	**25**	**18.9%**
Alone	**7**	
Combined	**18**	
***Acacia nilotica***	**13**	**9.8%**
Alone	**13**	
Combined	**0**	
***Cuminum cyminum***	**7**	
Alone	**2**	
Combined	**5**	**5.3%**
***Citrullus colocynthis***	**3**	
Alone	**3**	**4.5%**
Combined	**0**	
**Unknown Herbs used**	**66**	**50%**
**Different Other Herbs used**	**18**	**13.6%**

**Figure 1:**
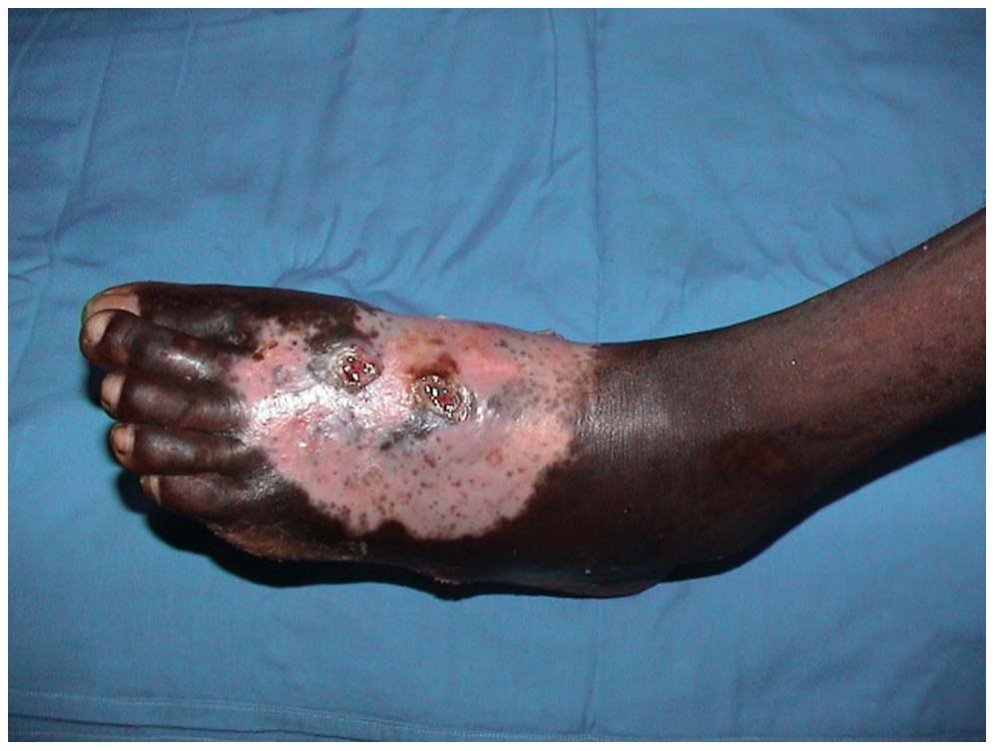
Showing massive foot mycetoma with skin burn following herb treatment.

The study population knowledge and opinion on herbal treatment are summarised in Table 3. Ninety one patients (29.3%) had encountered complications with herbal treatment and these were infection, burn and skin necrosis. (Fig. 1) Twenty patients (6.4%) who developed complications said that, they would inform their doctors about that only if they had been asked while five patients (1.6%) would deny that even if they had been asked about that.

**Table 3:** The study population knowledge and opinion on herbal treatment.

The Patients' knowledge & opinion	No. (%)
Did not know the meaning of herbal treatment	115 (37%)
Recognized it as natural products of plants and herbs	113 (36%)
Believed it is a traditional medicine	63 (20%)
Defined it as alternative medicine preparation with no added chemical.	12 (4%)
Believed that, the herbal treatment was not beneficial	59 (19%)
Reported partial improvement in their conditions	47 (15%)
Considered herbal treatment as a safe treatment modality.	53 (17%)
There was no significant association between the patients knowledge and	There was significant statistical association between the patients knowledge on mycetoma and
• Treatment duration (p = 0.08)	• Patients ages (p = 0.0)
• Mycetoma duration (p = 0.43)	

The studied patients were of different age groups, occupations, educational levels and different mycetoma knowledge levels. The study showed no significant statistical association between the herbal treatment use and the patients' education (*p* = 0.3), their ages (*p* = 0.6) and their knowledge on mycetoma (*p* = 0.1). There was statistically significant association between the herbal treatment use and the mycetoma duration (*p* = 0.037). (Table 1)

The patients' knowledge on the various aspects of mycetoma such as the clinical presentation, investigations and treatment was determined by questionnaire with Likert scale and classified as good knowledge (score of more than 10 points), moderate (5–10 points) and poor (less than 5 points). The study showed, most of the patients 177(56.9%) had moderate knowledge. There was significant statistical association between the patients knowledge on mycetoma and their ages (*p* = 0.0); the younger patients had better knowledge on mycetoma than the elder patients. Moreover, there was no significant association between the patients knowledge and treatment duration (*p* = 0.08) and mycetoma duration (*p* = 0.43). Table 3

*Moringa oleifera* was the commonly used herb, was documented in 25 patients (18.9%); 19 of them were in the age group 18–37 years, 11 of them had secondary school education and 17 of them had disease duration of more than 10 years. Data on the use of *Moringa oleifera* are shown in Table 4.

**Table 4:** The use of *Moringa oleifera* among the study population.

The use of Moringa oleifera	No. (%)
Used as a hot drink	15 (60%)
Used topically	6 (40%)
Used the Moringa oleifera leave	23 (92%)
Other parts of the tree.	2 (08%)
Used Moringa oleifera alone	7 (28)
Used it with other herbs	18 (72%)
There was no significant statistical association between the use of Moringa oleifera and	
• Patients ages (p = 0.1)	
• Mycetoma duration (p = 0.3)	
• Patients' occupation (p = 0.3	
• Patients knowledge on mycetoma (p = 0.2)	

## Discussion

The herbal treatment is a well-known treatment modality since the infancy of the humanity. It is used worldwide and in the Sudan, it is part of the local culture and religious beliefs (unpublished data). The WHO had estimated the prevalence of herbal and other non-conventional treatment as 70–80% [13]. In the Sudan, herbal treatment is more popular than the conventional treatment in many communities and that is due to social believes, the massive lack of health education, scarcity of medical and health facilities in remote areas, transport difficulties and financial constraints.

The objectives of this study were to determine the prevalence and knowledge of herbal treatment among a group of patients with mycetoma. Herbal treatment is commonly used in mycetoma, as the disease is one of the badly neglected tropical diseases, has a gradual onset and course and painless in nature in most of the patients, hence many patients present late with advanced disease. Furthermore the available medical and surgical treatment are unsatisfactory and expensive [8], [10]. As far as the authors know, this study is the first report on the herbal treatment of mycetoma.

The study showed a high prevalence of herbal treatment usage among the studied cohorts. The explanation of this is multifactorial, to mention but few, the patients poor socio-economical classes and health education and their dissatisfaction with the prolonged medical treatment, its many side effects and the high recurrent rate [8], [10].

The herbal treatment is characterized by wide availability, cultural acceptability, reasonable cost and the conception of safety among most patients. This study demonstrated a perception of safety in more than half of herbal users and this may explain the high prevalence of herbal treatment among the study population.

Many observations from the Sudan and from the current study showed that, this type of treatment is associated with many complications such as skin burn and necrosis, sepsis, septicaemia and it is an important cause for further local tissue damage and destruction, deformity and disability. It interesting to note that, many patients tend to deny these complications for various reasons.

Lack of governmental control and health policies that regulate herbal treatment in many countries and in Sudan in particularly may have contributed to the high prevalence of this modality among the studied patients. It is interesting to note, the WHO reported, the lack of national policy in 64% of the responded countries regarding the control of traditional medicine and complementary/alternative medicine [14].

Surprising, in this study, 66 (50%) of herbal treatment users did not know the neither the herb type nor its ingredients, this can be explained by the fact that most of these patients were simple persons and convinced by the traditional healers opinions and advices as most of healers are religious leaders as well.

The study showed a significant association between the prevalence of herbal treatment and the mycetoma duration. That is most probably due to the fact that patients with prolonged complicated disease are desperate and tent to try different therapeutic modalities as the current treatment outcome is unsatisfactory.

*Moringa oleifera* tree is commonly seen the tropics and subtropics regions and have numerous medicinal properties and recently if became a popular element in herbal treatment. Various parts of this plant such as the leaves, roots, seed, bark, fruit and flowers were tested and they showed activities as cardiac and circulatory stimulants, possess antitumor, antipyretic, anti-inflammatory, antispasmodic, diuretic, antihypertensive, cholesterol lowering, anti-diabetic, hepatoprotective, antibacterial and antifungal activities [15]–[21].

In the Sudan, there is a recent trend to use *Moringa oleifera* as herb for many medical problems; this could be attributable to the influence of media and its wide availability in the country. All these could explain the high prevalence of its use in this study.

In mycetoma, usually herbal treatment is used as local application in the lesion, but most of the studied patients used *Moringa oleifera* as a hot drink as it commonly used for other medical problems.

The study showed that, most of the patients were unsatisfied with herbal treatment they had, while few of them claimed some improvement, however, all these are subjective observations. Randomised clinical trials to study the effective and safety of herbal treatment in mycetoma are needed.

In conclusion, the study showed high prevalence of herbal treatment usage among the studied cohort. That can be explained by the patients' gross dissatisfaction of with the current medical therapeutic modalities which proved to be costly to patients, families and health authorities and have many side effects. The massive lack of health education among the patients, their low socio-economic status and scarcity of the medical and health facilities in endemic areas are the main causes of the high prevalence of the herbal treatment. Governmental control and health policies to control the herbal treatment are mandatory to reduce its prevalence. Native healers need to be educated to reduce the prevalence and complications of this treatment.

*Moringa oleifera* is the commonly used herb in this study, however, further in depth study to determine the active ingredients in the different parts of the tree and its effective is required.

## Supporting Information

Checklist S1 STROBE checklist.
(DOC)
